# Transgenic aspen and birch trees for Russian plantation forests

**DOI:** 10.1186/1753-6561-5-S7-P124

**Published:** 2011-09-13

**Authors:** Konstantin Shestibratov, Vadim Lebedev, Alexey Podrezov, Margarita Salmova

**Affiliations:** 1 Branch of Shemyakin and Ovchinnikov Institute of Bioorganic Chemistry RAS

## Background

Aspen(*Populus tremula*) and birch (*Betula pubescens*) are the fast-growing trees successfully used for the aim of plantation forestry. These species have the great potential in Russia to meeting the need for paper, timber and other wood-based products. However, enhanced growth rate, decreased lignin content and herbicide resistance are the required properties of new trees for plantation forestry. Breeding of forest trees is a slow process due to the long generation intervals typical of most forest trees and because many traits can only be properly assessed at rotation age. Genetic modification is an alternative method that can be used for new trees creation.

## Methods

Genetic transformation experiments were carried out using vectors pBI-4CL, pBI-Xeg, pGS and pBIBar and supervirulent *Agrobacterium tumefaciens* strain CBE21. pBI-4CL plasmid contains the expression cassette harboring inverted fragments of 4-coumarat-CoA-ligase gene (GeneBank AY043494). pBI-Xeg vector was constructed using cDNA of xyloglucanase gene cloned from *Penicillium canescens*. pGS and pBIBar vectors contains pine glutamine synthetase gene (*GS*) and phosphinothricin acetyltransferase gene (*bar*). For transcription control of expression 35S promoter used in all cassettes harboring the gene of interest.

Internodes from *in vitro* aspen plants (*Populus tremula*) and leaves from *in vitro* birch plants (*Betula pubescens*) cultivating on WPM medium were used as an explants for *Agrobacterium*-mediated transformation [1,2]. Transformed shoots selected on the modified MS medium with 25 (aspen) or 50 (birch) mg/l kanamycin.

To detect DNA fragments from binary vectors transformants were analysed by PCR. Expression of recombinant genes was detected by RT-PCR. Lignin content determination performed by using the Klason method.

## Results and conclusions

**Figure 1:**
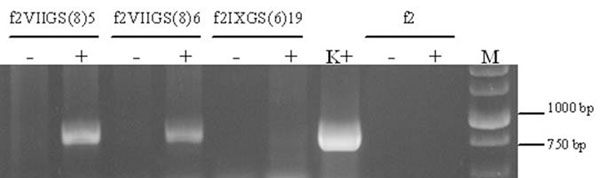
RT-PCR analysis of a glutamine synthetase gene expression in transgenic aspen plants: + and – are RNA preparations with or without the treatment with reverse transcriptase, respectively; K+ is pGS plasmid; f2 is nontransformed control; M - molecular marker.

As a result of two *Agrobacterium*-mediated transformation experiments with pGS vector 56 and 27 transgenic lines of birch and aspen have been produced respectively. Presence of recombinant glutamine synthetase *GS* gene confirmed by PCR in 52 birch and 23 aspen lines. *GS* gene expression at the RNA level demonstrated RT-PCR analysis (Figure 1).

Most of GS lines have resistance to subletal dose of phosphinotricin as well as in the in vitro and greenhouse conditions. Comparative biometric analysis of GS plants in a greenhouse makes it possible to select 7 and 3 superior lines of birch and aspen respectively. Growth rate enhancement of selected lines was 20-25 %.

**Figure 2:**
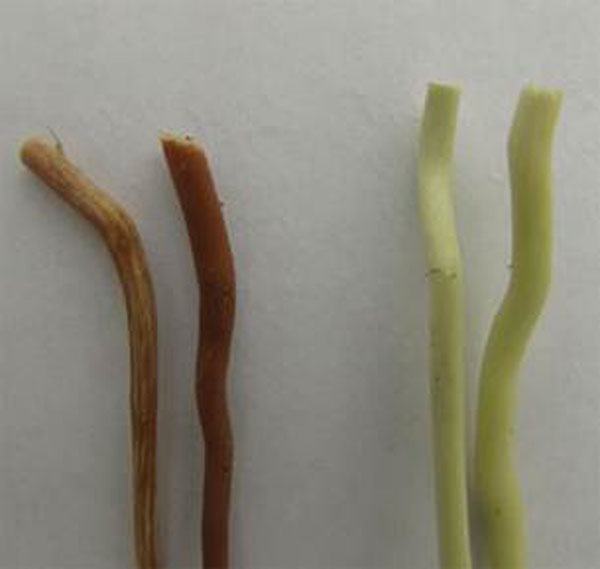
Stem wood coloration of control (right) and transgenic 4CL plants (left).

Genetic transformation of aspen plants for 4-coumarat-CoA-ligase gene down-regulation and xyloglucanase overexpression has been performed using pBI-4CL and pBI-Xeg binary vectors respectively. 11 transgenic lines with inverted fragments of 4CL gene and 15 lines harboring Xeg1 gene have been produced. DNA fragments of 4CL RNAi-construct were detected in all lines. 14 out of 15 transformants produced with pBI-Xeg vector harboring Xeg1 gene. Chemical analysis of stem wood of 4CL lines demonstrated decreased lignin content (Table 1). Lignin reduction from 11 to 23 % was achieved. Line PtXIII4CL2cdemonstrated minimum lignin content 18,7 %. Color modification of stem wood was observed for 8 out of 11 4CL lines (Figure 2).

**Table 1:** Lignin content of extract-free wood from control and transgenic aspen plants.

Line	Acid soluble lignin, %	Acid insoluble lignin, %	Total lignin, %	Lignin reduction, %
Pt (control)	21,0	3,4	24,4	-
PtXIII4CL1c	21,4	3,0	24,3	0,2
PtXIII4CL2c	15,2	3,6	18,7	23,2
PtXIII4CL3a	22,5	3,0	25,5	-4,7
PtXIII4CL3c	17,9	3,7	21,7	11,1
PtXIII4CL4a	18,5	3,2	21,7	11,1
PtXIII4CL4c	17,4	3,8	21,2	13,0

Aspen and birch transgenic plants with *bar* gene coding resistance against herbicide phosphinotricin were also created. Transformation experiments were carried out using vector pBIBar. 19 aspen and 9 birch lines were selected as a result of transformation. Presence of *bar* gene was confirmed in all lines. Herbicide resistance was observed at 16 and 7 lines of aspen and birch respectively. Lines tested in a greenhouse demonstrated normal phenotype without somaclonal variations and may be recommended for the field trials.

To combine several new properties in one plant the co-transformation method was developed. This method allows transferring recombinant DNA fragments from three T-DNAs of two plasmids independently [3]. Efficiency of co-transformation was up to 100 %. Currently we attempt to apply this method for the production of superior aspen and birch plants with enhanced growth rate, decreased lignin content and herbicide resistance at the same time.
